# Age, comorbidity burden and late presentation are significant predictors of hospitalization length and acute respiratory failure in patients with influenza

**DOI:** 10.1038/s41598-024-66550-8

**Published:** 2024-07-06

**Authors:** Victor Daniel Miron, Oana Săndulescu, Anca Streinu-Cercel, Dragoș Florea, Simona Paraschiv, Leontina Bănică, Ovidiu Vlaicu, Dan Oțelea, Anuța Bilașco, Daniela Pițigoi, Adrian Streinu-Cercel, Anca Cristina Drăgănescu

**Affiliations:** 1 Carol Davila University of Medicine and Pharmacy, Bucharest, Romania; 2 National Institute for Infectious Diseases “Prof. Dr. Matei Balș”, Bucharest, Romania

**Keywords:** Influenza, Hospitalization, Children, Adults, Elderly, Acute respiratory failure, Health care, Risk factors, Signs and symptoms

## Abstract

Influenza viruses are responsible for a high number of infections and hospitalizations every year. In this study, we aimed to identify clinical and host-specific factors that influence the duration of hospitalization and the progression to acute respiratory failure (ARF) in influenza. We performed an analysis of data from a prospective active influenza surveillance study that was conducted over five seasons (2018/19 to 2022/23). A total of 1402 patients with influenza were included in the analysis, the majority of which (64.5%) were children (under 18 years), and 9.1% were elderly. At least one chronic condition was present in 29.2% of patients, and 9.9% of patients developed ARF. The median hospital stay was 4 days (IQR: 3, 6 days). The most important predictors of prolonged hospital stay and development of ARF were extremes of age (infants and elderly), presence of chronic diseases, particularly the cumulus of at least 3 chronic diseases, and late presentation to hospital. Among the chronic diseases, chronic obstructive pulmonary disease, cardiovascular disease, cancer, diabetes, obesity, and chronic kidney disease were strongly associated with a longer duration of hospitalization and occurrence of ARF. In this context, interventions aimed at chronic disease management, promoting influenza vaccination, and improving awareness and access to health services may contribute to reducing the impact of influenza not only in Romania but globally. In addition, continued monitoring of the circulation of influenza viruses is essential to limit their spread among vulnerable populations.

## Introduction

Every cold season, influenza viruses pose important health challenges, causing high numbers of human infections, with clinical pictures ranging from mild to severe. Many patients with influenza may require hospitalization and even admission to the intensive care unit (ICU)^1^. The European Centre for Disease Prevention and Control^2^ and the US Centers for Disease Control and Prevention^3^ have defined a set of patient groups who are at higher risk of unfavorable influenza outcomes, particularly elderly people and persons with chronic conditions. At the same time, a number of influenza-specific severity scores have been developed (IDEAS scale^4^, FluA-p score^5^, FLU-PRO^6^), but most remain underused, as their implementation can sometimes be challenging for the clinician due to the inclusion of either laboratory parameters or a large number of variables. In addition, the spread and impact of influenza virus infection is also influenced by the characteristics of the population (individual and socio-economic) in a given geographical area/region^7–9^. Furthermore, knowledge of regional data is important to establish appropriate management and tailored strategies to prevent and control potential influenza epidemics or pandemics.

Romania experiences a high number of influenza cases each year, and vaccination coverage is low, reported at only 23% among the elderly and 8% among children and healthcare workers in the 2022/23 season^10^. Understanding the specific characteristics of patients with influenza in this region and identifying risk factors that could potentially be assessed right from the initial medical assessment is essential. This will allow early therapeutic decisions to be made and complications to be prevented in those identified as being at risk.

In this context, through a comprehensive analysis of data from active influenza surveillance studies in Romania, we aimed to identify clinical and host-specific factors that influence the duration of hospitalization and progression to acute respiratory failure (ARF) in influenza virus infection.

## Methods

We conducted an analysis of data from prospective active influenza surveillance studies conducted at the National Institute of Infectious Diseases "Prof. Dr. Matei Balș" (NIID), Bucharest, over five consecutive seasons. NIID is the largest hospital for infectious diseases (covering children and adults) in Romania and is part of the international influenza active surveillance networks Global Influenza Hospital Surveillance Network (GIHSN) and Development of Robust and Innovative Vaccine Effectiveness (DRIVE, 2018 to 2022).

The methodology of the studies together with the patient selection criteria and the protocol for identification of influenza viruses by RT-PCR have been fully described in previously published reports^11–14^. In brief, patients hospitalized during an influenza season (usually November–May) for influenza-like illness (ILI) or severe acute respiratory infection (SARI) with symptom onset of 7 days or less and who consented to participate in the study were tested by RT-PCR for the influenza viruses and were followed-up for influenza-related outcomes. Clinical data were collected through a standardized medical questionnaire.

We included in the analysis data from all patients hospitalized for influenza, who were enrolled in active influenza surveillance studies over five seasons (2018/19 to 2022/23). Demographic (sex, age), clinical (symptoms of ILI and SARI^15^), chronic conditions (cardiovascular disease, chronic obstructive pulmonary disease (COPD), asthma, other chronic lung disease, immune disease, rheumatological disease, diabetes, chronic kidney disease (CKD), obesity, cancer, chronic liver disease, neurological disease, HIV infection and other chronic conditions) and clinical outcomes (requirement of oxygen supplementation, intensive care admission, mechanical ventilation, occurrence of pneumonia) were assessed to identify predictors of length of hospital stay and progression to acute respiratory failure. The following age groups were defined: infant (0–11 months), toddler (1–2 years), preschooler (3–4 years), schooler (5–13 years), teenager (14–17 years), adult (18–64 years), elderly (65 years and over).

Statistical analysis was performed using IBM SPSS Statistics for Windows, version 25 (IBM Corp., Armonk, NY, USA). The chi-squared test (χ^2^) with risk calculation by odds ratio (OR) and 95% confidence interval (95%CI) was used to analyze categorical variables. Logistic regression tests were used to stratify variables with statistical significance. For continuous variables, the Shapiro–Wilk test was used to check the normality of the distribution. As all our variables were non-parametrically distributed, we present the median and interquartile range (IQR), while differences between groups were analyzed using the Mann–Whitney U test (U) and the Kruskal–Wallis H test (H). The effect size (r) for the two tests was calculated as described in the literature^16^. The level of statistical significance was set at p < 0.05.

The study was approved by the Bioethics Committee of the National Institute for Infectious Diseases “Prof. Dr. Matei Balș”, Bucharest, Romania. The study was conducted in accordance with the Declaration of Helsinki. All patients or legal guardians, as appropriate, provided written informed consent prior to performing any study-related procedures.

## Results

### Patient characteristics

A total of 1402 patients with influenza were included in the analysis, the majority of which (64.5%, n = 904) were children (under 18 years), and 9.1% (n = 128) were elderly. The median age was 7.6 years (IQR: 2.6, 33.4 years) and there was a slight predominance of females (52.5%, n = 736).

Approximately one third of patients had at least one chronic disease (29.2%, n = 409). In terms of influenza outcomes, 9.9% (n = 139) had ARF requiring oxygen supplementation, and 3.5% (n = 49) required admission to ICU. All characteristics of the patients included in the study are shown in Table 1.

**Table 1:** General characteristics of patients included in the study.

Characteristics	Frequency (N = 1402)
Female, n (%)	736 (52.5)
Age, median (IQR)	7.6 years (IQR: 2.6, 33.4 years)
Infants, n (%)	161 (11.5)
Toddlers, n (%)	220 (15.7)
Preschoolers, n (%)	167 (11.9)
School children, n (%)	314 (22.4)
Teenagers, n (%)	42 (3.0)
Adults, n (%)	370 (264)
Elderly, n (%)	128 (9.1)
Pregnancy (for females), n (%)	21/419* (5.0)
Chronic conditions (at least one), n (%)	409 (29.2)
3 or more chronic conditions, n (%)	132 (9.4)
Cardiovascular disease, n (%)	175 (12.5)
Chronic obstructive pulmonary disease, n (%)	30 (2.1)
Asthma, n (%)	36 (2.6)
Other chronic lung disease, n (%)	12 (0.9)
Diabetes mellitus, n (%)	72 (5.1)
Immune disease, n (%)	39 (2.8)
Chronic kidney disease, n (%)	39 (2.8)
Rheumatological disease, n (%)	28 (2.0)
Neurological disease, n (%)	47 (3.4)
Chronic liver disease, n (%)	49 (3.5)
Cancer, n (%)	47 (3.4)
Obesity, n (%)	89 (6.3)
HIV infection, n (%)	27 (1.9)
Other chronic disease, n (%)	138 (9.8)
Influenza vaccine, n (%)	66 (4.7)
Days from onset of symptoms, median (IQR)	2 days (IQR: 1, 3 days)
Onset of symptoms more than 3 days, n (%)	262 (18.7)
Acute respiratory failure, n (%)	139 (9.9)
Mechanical ventilation, n (%)	14 (1.0)
Intensive care unit admission, n (%)	49 (3.5)
Viral pneumonia, n (%)	96/421^#^ (22.8)
Co-infections, n (%)	75 (5.3)

*Data available for 419 fertile women; ^#^ data available from last 2 seasons for 421 patients; infants: 0–11 months; toddlers: 1–2 years; preschoolers: 3–4 years; schoolers: 5–13 years; teenagers: 14–17 years; adults: 18–64 years; elderly: 65 years and over.

**Figure 1:**
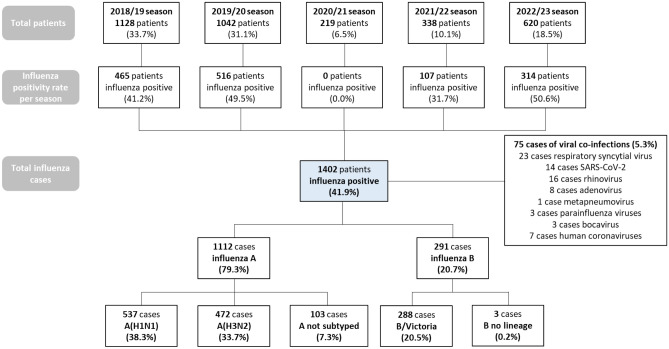
Study chart with distribution of cases by seasons.

Influenza A was the predominant viral type (79.3%, n = 1112), especially the influenza A(H1N1) subtype (38.3%, n = 537). Co-infections with other respiratory viruses were identified in 75 cases (5.3%) (Fig. 1).

### Predictors of length of hospitalization

**Figure 2:**
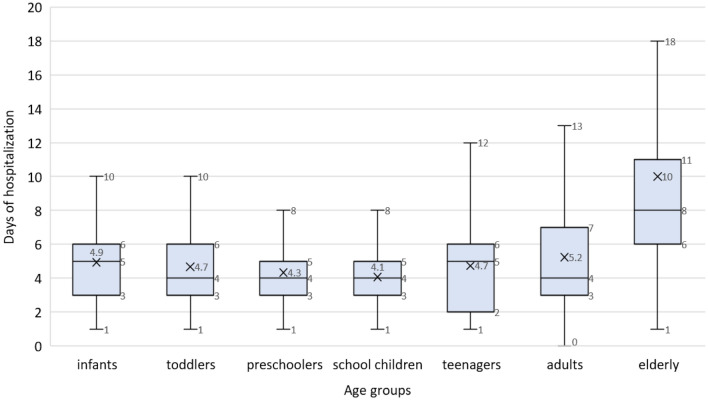
Distribution of the median length of hospitalization by age group. Infants: 0–11 months; toddlers: 1–2 years; preschoolers: 3–4 years; schoolers: 5–13 years; teenagers: 14–17 years; adults: 18–64 years; elderly: 65 years and over.

Overall, the median length of hospital stay was 4 days (IQR: 3, 6 days) and was not influenced by gender (same value for both sexes, p = 0.673), but it varied by age group. Elderly patients required up to 8 days (IQR: 6, 11 days) of hospitalization, whereas infants had a median of 5 days (IQR: 3, 6 days) (p < 0.001, H(6) = 136.6). The distribution of the length of hospital stay by age group is shown in Fig. 2. The median duration of hospital stay for a patient with influenza without complications or risk factors was 2 days (IQR: 1, 3 days), p < 0.001.

Clinically, only the presence of dyspnea and deterioration of the general condition at the time of admission were significantly associated with increased length of hospital stay, up to 7 days (IQR: 4, 10 days) p < 0.001 for dyspnea, and up to 5 days (IQR: 3, 7 days) p < 0.001 for deterioration. The other analyzed symptoms did not influence the length of hospital stay (p > 0.05 for each). Admission to hospital more than 3 days after symptom onset was associated with a median length of hospital stay increased to 5 days (IQR: 3, 7 days), p = 0.001.

The presence of at least one chronic condition was associated with a median length of stay of 6 days (IQR: 4, 8 days), p < 0.001, while the cumulative association of at least three chronic conditions increased the length of stay to 8 days (IQR: 6, 11 days), p < 0.001. Among the chronic conditions, cardiovascular disease, COPD, obesity, diabetes, cancer, CKD, chronic liver disease, and neurological disease significantly increased the length of hospital stay (Table 2).

**Table 2:** Length of hospitalisation according to patient characteristics.

Characteristics	Length of hospitalization (days)			p-value	Z value	Effect size (r)
	Median	Q1 (25%)	Q3 (75%)			
Chronic conditions						
At least one chronic disease	6	4	8	< 0.001	− 13.496	0.360
3 or more chronic diseases	8	6	11	< 0.001	− 7.612	0.203
Cardiovascular disease	7	5	10	< 0.001	− 11.890	0.317
Chronic obstructive pulmonary disease	8	7	11	< 0.001	− 6.408	0.171
Asthma	5	3	7	0.077	− 1.879	0.050
Other chronic lung disease	7	5	10	0.042	− 2.031	0.054
Diabetes mellitus	7	5	10	< 0.001	− 7.059	0.188
Immune disease	7	4	11	< 0.001	− 5.317	0.142
Chronic kidney disease	9	6	14	< 0.001	− 7.192	0.192
Rheumatological disease	6	5	11	< 0.001	− 4.019	0.107
Neurological disease	6	4	8	< 0.001	− 4.334	0.115
Chronic liver disease	6	5	8	< 0.001	− 6.557	0.175
Cancer	7	5	11	< 0.001	− 5.717	0.152
Obesity	6	4	8	< 0.001	− 5.385	0.143
HIV infection	6	3	7	0.056	− 1.914	0.051
Other chronic disease	5	4	7	0.032	− 5.419	0.144
Clinical features						
Acute respiratory failure	8	6	12	< 0.001	− 13.137	0.350
Viral pneumonia	5.5	4	8	< 0.001	− 6.698	0.178
Intensive care unit admission	8	6	13.5	< 0.001	− 7.408	0.197
Mechanical ventilation	11.5	7	19	< 0.001	− 4.296	0.115
Influenza vaccine	4	3	7	0.561	− 0.581	0.015
Co-infections						
Influenza A(H1N1)	4	3	6	0.051	− 1.154	0.030
Influenza A(H3N2)	4	2	6	0.041	− 1.320	0.035
Influenza A not subtyped	5	3	7	0.073	− 1.794	0.048
Influenza B/Victoria	4	3	6	0.147	− 1.452	0.038
Co-infections	5	3	7	0.452	− 0.753	0.020
SARS-CoV-2	5	4	7	0.020	− 2.330	0.114
Respiratory syncytial virus	6	3	9.5	0.046	− 1.993	0.053
Rhinovirus	4	2	6	0.659	− 0.441	0.021

SARS-CoV-2—severe acute respiratory syndrome coronavirus 2.

The occurrence of viral pneumonia prolonged the duration of hospitalization by 1.5 days (5.5 days (IQR: 4, 8 days), p < 0.001) and the presence of ARF by 4 additional days (8 days (IQR: 6, 12 days), p < 0.001). Similarly, a longer duration of hospitalization was noted for patients admitted to the ICU (8 days (IQR: 6, 13.5 days), p < 0.001) and in particular those requiring mechanical ventilation (11.5 days (IQR: 7, 19 days), p < 0.001).

We found no differences in length of hospital stay according to influenza virus type and subtype/lineage. Among co-infections, only respiratory syncytial virus (RSV) and SARS-CoV-2 had a significant effect on length of hospital stay (Table 2).

### Predictors of acute respiratory failure

A total of 139 (9.9%) patients with influenza developed ARF. This occurred in all age groups, but was most prevalent in the elderly, who had an 8.9-fold increased risk (p < 0.001, χ^2^(1) = 141.3, OR = 8.9, 95%CI 5.9–13.5) of developing respiratory failure compared with other age groups. The median age of those with ARF was 57.3 years (IQR: 5.3, 71.0 years).

**Figure 3:**
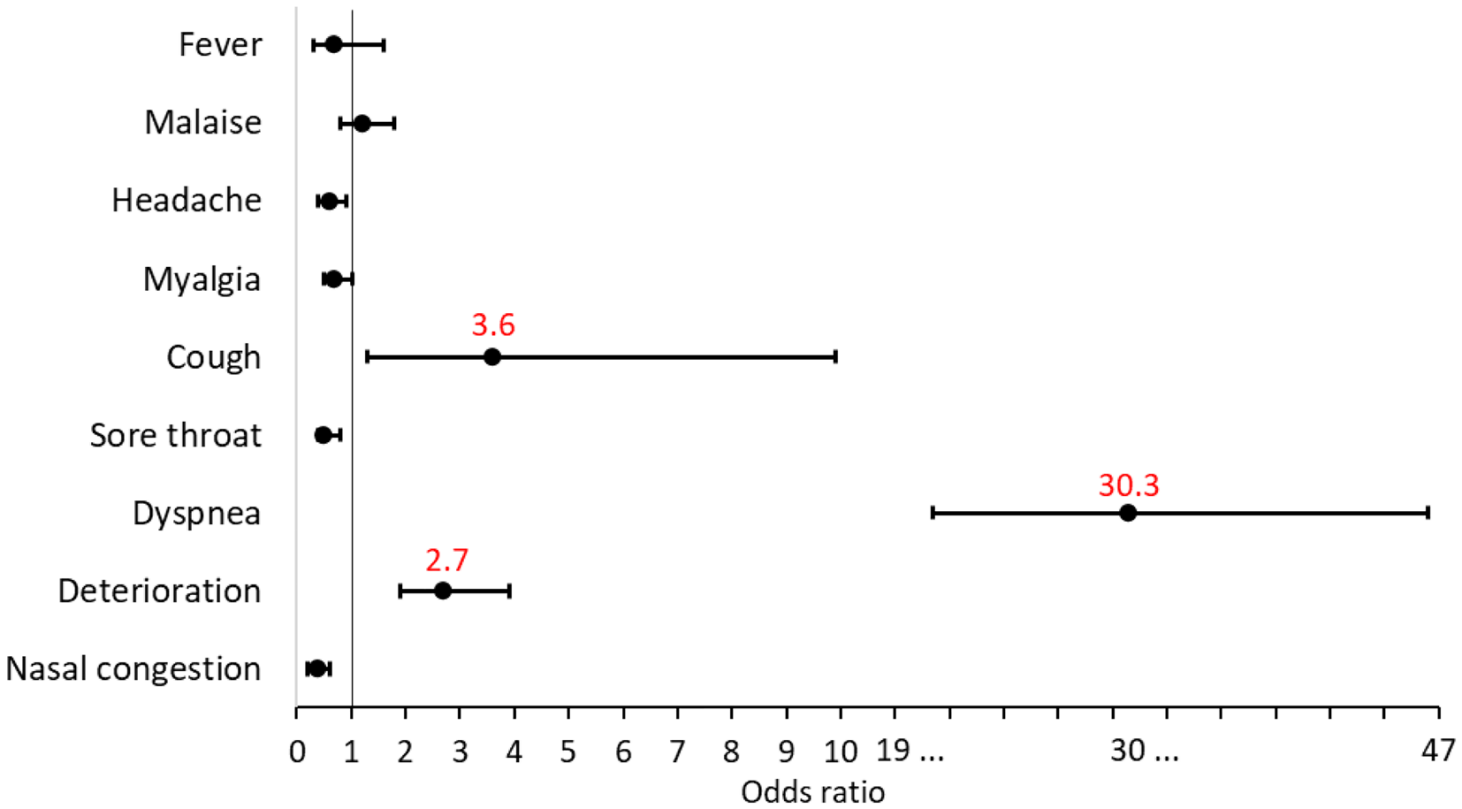
Probability of predicting progression to acute respiratory failure based on symptoms at hospital admission.

Clinically, the presence of dyspnea was the best predictor of progression to ARF (OR = 30.3, p < 0.001). Other symptoms associated with an increased likelihood of ARF were cough (OR = 3.6, p = 0.008) and deterioration of the general condition (OR = 2.8, p < 0.001), while nasal congestion (OR = 0.4, p < 0.001), headache (OR = 0.6, p = 0.018) and odynophagia (OR = 0.5, p = 0.002) decreased the likelihood of ARF if present at the time of hospital admission (Fig. 3, Table 1S supplementary material).

**Figure 4:**
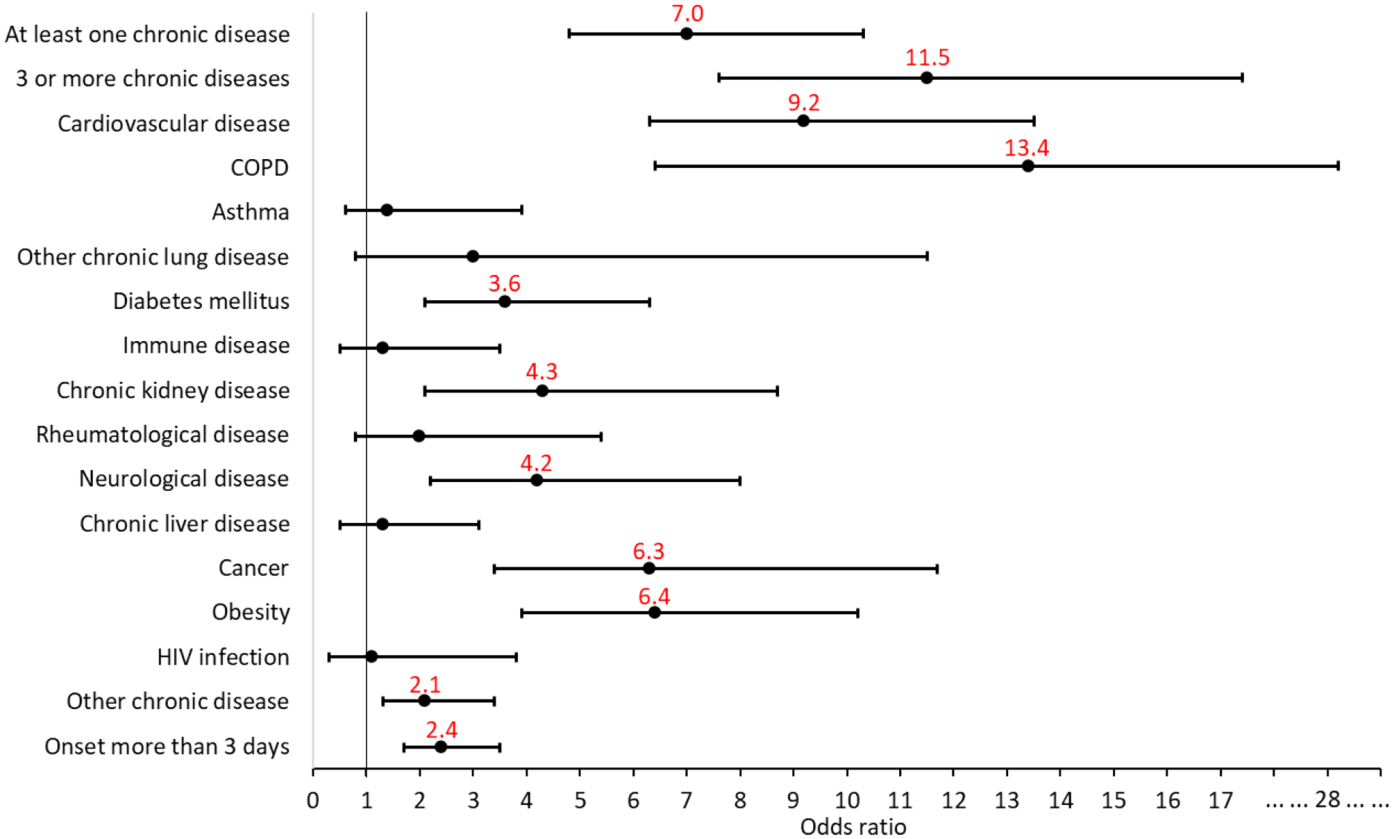
Probability of predicting progression to acute respiratory failure based on chronic conditions. COPD—chronic obstructive pulmonary disease.

Chronic conditions were strongly associated with ARF. The presence of at least one chronic disease was associated with a sevenfold increase in risk (p < 0.001), while the association of at least three chronic diseases increased the risk 11.5-fold (p < 0.001). COPD, cardiovascular disease, cancer and obesity were most strongly associated with progression to ARF. We also observed that late presentation to the hospital, more than 3 days after symptom onset, increased the risk of respiratory failure 2.4 times (p < 0.001; Fig. 4, Table 1S supplementary material).

**Figure 5:**
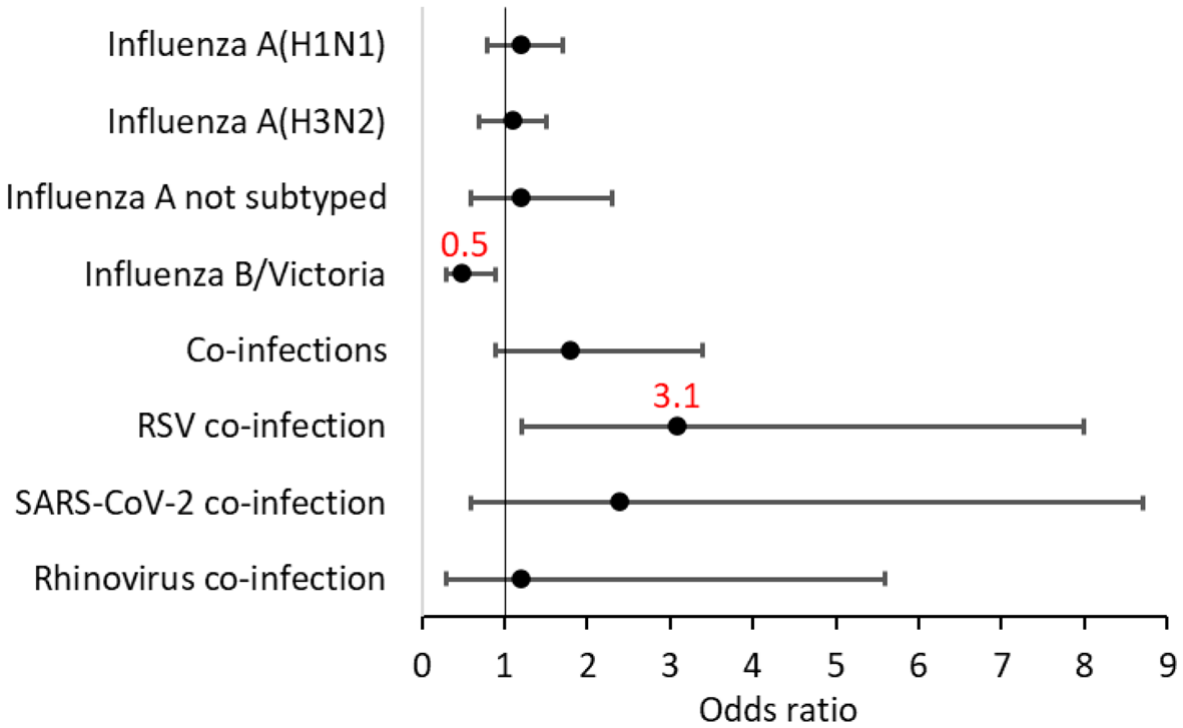
Probability of predicting progression to acute respiratory failure according to type of influenza virus and co-infections. RSV—respiratory syncytial virus; SARS-CoV-2—severe acute respiratory syndrome coronavirus 2.

Influenza B/Victoria cases had twofold lower odds (OR = 0.5, p = 0.019) of progression to ARF compared to influenza A cases (Fig. 4). Conversely, influenza A cases carried higher odds of progression to ARF compared to influenza B (OR = 1.6, p = 0.053). The presence of viral co-infections appeared to more frequently lead to ARF (8.6% vs. 5.0%, p = 0.070); in particular, RSV co-infection was associated with a 3.1-fold increased risk (p = 0.013, Fig. 5, Table 1S supplementary material).

Throughout the 5 influenza seasons analyzed, 12 deaths were recorded in the study group, with an overall case fatality rate of 0.9%. All deaths occurred in adult patients, who had influenza A, and who had developed ARF.

## Discussion

Through this analysis of data from prospective active influenza surveillance studies, we aimed to provide a comprehensive picture of the interplay between patient characteristics and influenza outcomes, in order to identify factors influencing length of hospital stay and progression to ARF in patients with influenza in Romania. These results may be useful for the effective management of influenza cases by providing rapid decision support to clinicians.

We found a median hospital stay of 4 days, which was reduced to 2 days in patients without risk factors and/or co-infections. In fact, this is the period during which the etiological diagnosis is established, the severity of the disease is assessed, antiviral treatment is initiated, its tolerability is evaluated and, in patients without additional risk factors, a first therapeutic response is achieved, generally with rapid remission of fever and general symptoms. In comparison, the median length of hospital stay at the extremes of age was 5 days for infants and 8 days for the elderly, highlighting the fact that these age groups are disproportionately affected by influenza and drawing attention to the burden on the healthcare system and the patient, caused by a vaccine-preventable disease. Therefore, regardless of the cumulative presence of other risk factors, efforts must be made to increase influenza vaccination rates in these populations. This is of particular importance in Romania where, as mentioned in the introduction, the influenza vaccination coverage for the most recent influenza season (2022/23) was 23% among the elderly^10^. In contrast, in the United States, the average vaccination coverage for the same age group in the same season was 69.7%^17^, much closer to the 75% target of the World Health Assembly and the European Council.

A number of very interesting results from this study identify the main host characteristics associated with prolonged hospitalization. Of these, the presence of a chronic disease had the greatest impact (median length of stay 6 days), but especially the cumulative presence of at least three chronic diseases (8 days). Specifically, COPD (8 days), CKD (9 days), cardiovascular disease (7 days), cancer (7 days), diabetes (7 days) and obesity (6 days) had the greatest impact on length of stay. It is important to note that these are the most common comorbidities found in patients with influenza but also in the general population. Analysis of data from the 2019/20 season from all GIHSN study centers, which applied the same protocol as our study, identified a prevalence of 28.2% for cardiovascular disease, 9.2% for COPD, 11.8% for diabetes, 6.9% for CKD and 8.7% for obesity across 19 centers with a total of 3021 influenza patients^18^. Most of these percentages are lower than those found in our study in Romania, which can be explained by the demographic diversity of the populations of the countries included in the GIHSN analysis. Clearly, the health status of a country's population and the prevalence of chronic diseases in that population have an important impact on the clinical outcome of influenza. Public health policies should be aimed at preventing and controlling influenza epidemics while also focusing on preventing and controlling chronic diseases, which are important risk factors for adverse outcomes of influenza, as we have shown in our study. Reducing the incidence and prevalence of chronic diseases will modulate the negative impact of influenza, even in the face of a pandemic. In a study from Canada, Kim P. et al. showed that the burden of severe influenza in people over 50 years of age remains significant despite publicly funded vaccination programmes, as long as the prevalence of chronic diseases increases in the same population^19^.

A particularly important finding of our study relates to the precise identification of highly predictive factors for the development of ARF in influenza, namely: older patient age (8.9-fold increased risk), presence of a chronic condition (7.0-fold increased risk) or a combination of comorbidities (11.5-fold increased risk), in particular obesity (6.4-fold increased risk), cancer (6.3-fold increased risk), cardiovascular disease (9.2-fold increased risk), CKD (4.3-fold increased risk) and COPD (13.4-fold increased risk). The excess risk seen in patients with COPD is likely to be multifactorial. On the one hand, influenza is an infectious injury that occurs in the already fragile respiratory system of patients with chronic lung disease and can easily progress to severe forms with respiratory failure. On the other hand, influenza can lead to exacerbation of COPD, with loss of control of the chronic lung disease and the need for adjustment of comorbidity treatment, which is often a lengthy process and prolongs hospitalization beyond the actual influenza episode^20^. A very important issue to discuss is the impact of obesity on the adverse course of influenza. In a context where the prevalence of obesity is increasing worldwide, including in Romania^21^, being overweight can be a determining factor in increasing the burden of influenza. It is currently known that obesity is associated with a chronic pro-inflammatory status^22^, leading to pulmonary meta-inflammation^23^ with increased alveolar-capillary permeability and decreased epithelial regeneration^24^, which will lead to the development of ARF and increased length of hospitalization. In addition, it has been observed that obese patients with influenza have a prolonged persistence of viable influenza viruses in the airways, leading to increased infectivity, but also to a greater likelihood of accumulation of mutations in the viral genome^25^.

It is particularly important to understand and be aware of these host-specific risk factors, both for prolonged hospitalization and for severe forms of influenza with associated ARF. It is essential that medical education is provided to these groups of patients, and to the physicians of different specialties who care for them, to make patients aware of their specific risk and thus increase vaccination uptake.

Another particularly important element of our analysis is the identification of late presentation to the hospital as a major risk factor for prolonged hospitalization and the development of ARF. The importance of early diagnosis of influenza, with or without specific medical evaluation, correlates with the optimal interval for early initiation of antiviral treatment. In influenza, it is optimal to start treatment within the first 48 h of clinical onset. A study of eight consecutive influenza seasons showed that early administration of oseltamivir within the first 48 h reduced influenza mortality by 31%, including in critically ill patients^26^. In essence, late diagnosis and late presentation to the doctor miss out on the window of opportunity for this early initiation of antiviral treatment and allow viral and inflammatory damage associated with ARF and other specific complications to set in. It is therefore important to increase the referral of patients with influenza-like symptoms to primary care services for early diagnosis and treatment. It is also important to note that late presentation is not a contraindication to antiviral treatment; on the contrary, treatment should be initiated at any time, as soon as possible after the patient's initial assessment, particularly in severe cases^26^. A study conducted in France during the 2018/19 season raised a red flag about the misinterpretation of the recommendation for early antiviral treatment in influenza. Specifically, Cizeron et al.^27^ showed that oseltamivir was prescribed in only 59.9% of patients in their center and, paradoxically, less often in patients with risk factors (50%) than in those without risk factors (70%). Even more concerning was the rate of antiviral treatment administered in only 45.3% of severe influenza cases, and the main element identified by the authors as statistically significantly associated with inadequate prescription of antiviral treatment was late presentation of patients, within 48–120 h (median 72 h)^27^.

When analyzing influenza virus types, length of hospital stay was not significantly associated with any of the viral subtypes in our study, but odds of progression to ARF were higher for influenza A compared to influenza B. Conversely, infection with influenza B/Victoria viruses was associated with lower odds of progression to ARF (OR = 0.5). This may be an artificial finding, potentially due to the fact that 82.5% of the patients with influenza B in our analysis were children, a patient population that generally displays better outcomes of influenza, compared to elderly comorbid patients. This aspect of a predominant circulation of influenza B viruses in the pediatric population has also been observed worldwide^28^. However, overall, 16.8% of patients with ARF had influenza B, similar to a 6-year report showing that 20% of cases of respiratory distress due to influenza were caused by influenza B viruses^29^. Cases of respiratory failure associated with influenza B have been described in the elderly^30^, adults^31^ and children^32^ without associated chronic diseases. Therefore, the impact of influenza B should not be underestimated, as cases with an unfavorable outcome may occur even in patients without underlying risk factors. An anatomopathological review by Paddock et al.^33^ showed that patients who died from influenza B infection, whether or not associated with bacterial pneumonia, had a rapid clinical progression, with approximately half of all patients dying within 3 days of illness onset. Genetic factors may also play an important role in the progression of such cases, which is why the study of host specificities in relation to viral specificities is important in order to understand the pathophysiology of these interactions.

Our study analyzes data from active influenza surveillance according to standardized international protocols. However, it also has a number of limitations. First, the period analyzed (2018–2023) overlaps to a certain extent with the COVID-19 pandemic, which had a significant impact on influenza virus circulation and patient access to healthcare services. Second, a large proportion of the patients in our analysis were children. This is partly due to the fact that our institute is the main infectious disease hospital in the region, and many pediatric cases are referred to our hospital. Furthermore, hospitalization of children is also influenced by parental concerns and difficulties in home-based symptom management, and it might not always be a reflection of the true severity of the disease. However, the large number of patients and the extensive data analysis provide information that can easily be used in medical practice, which constitute important strengths of our current study.

## Conclusions

Our analysis showed that extremes of age (infants and the elderly), the presence of chronic conditions (especially COPD, cardiovascular disease, CKD, obesity, cancer and diabetes) and late presentation to the hospital have a significant impact on the length of hospital stay and the likelihood of developing acute respiratory failure during influenza virus infection. In this context, interventions aimed at managing chronic diseases, promoting influenza vaccination, and improving awareness of and access to health services can significantly contribute to reducing the impact of influenza. In addition, continuous monitoring of influenza virus circulation is essential to limit its spread among vulnerable populations.

### Supplementary Information

Supplementary Table 1S.﻿

## Data Availability

The datasets generated and analyzed during the current study are available from the corresponding author upon reasonable request.
