# Supplementary material for: Age, comorbidity burden and late presentation are significant predictors of hospitalization length and acute respiratory failure in patients with influenza
Source: Sci Rep. 2024 Jul 6;14:15563. doi: 10.1038/s41598-024-66550-8 (PMC11227496; doi:10.1038/s41598-024-66550-8)
Supplement: Supplementary file 1 — Supplementary Table 1S.﻿ [file 41598_2024_66550_MOESM1_ESM.docx]

**Table 1S (supplementary material). Analysis of the role of patient characteristics in predicting the risk of progression to acute respiratory failure**

| **Characteristics** | **With ARF, N=139, n(%)** | **Without ARF,**  **N=1263, n(%)** | **p-value** | **OR** | **95%CI** |
| --- | --- | --- | --- | --- | --- |
| Male | 62 (44.6) | 604 (47.8) | 0.471 | 0.9 | 0.6-1.2 |
| Female | 77 (55.4) | 659 (52.2) |  |  |  |
| Infants | 9 (6.5) | 152 (12.0) | 0.050 | 0.5 | 0.3-1.01 |
| Toddlers | 21 (15.1) | 199 (15.8) | 0.841 | 1.1 | 0.6-1.7 |
| Preschoolers | 4 (2.9) | 163 (12.9) | <0.001 | 0.2 | 0.1-0.5 |
| School children | 12 (8.6) | 302 (23.9) | <0.001 | 0.3 | 0.2-0.6 |
| Teenagers | 1 (0.7) | 41 (3.2) | NA | NA | NA |
| Adults | 41 (29.5) | 329 (26.0) | 0.380 | 1.2 | 0.8-1.7 |
| Elderly | 51 (36.7) | 77 (6.1) | <0.001 | 8.9 | 5.9-13.5 |
| Pregnancy | 0 (0.0) | 21 (1.7) | NA | NA | NA |
| Fever | 132 (95.0) | 1216 (96.3) | 0.445 | 0.7 | 0.3-1.6 |
| Malaise | 109 (78.4) | 949 (75.1) | 0.394 | 1.2 | 0.8-1.8 |
| Headache | 45 (32.4) | 540 (42.8) | 0.018 | 0.6 | 0.4-0.9 |
| Myalgia | 41 (29.5) | 474 (37.5) | 0.062 | 0.7 | 0.5-1.02 |
| Cough | 135 (97.1) | 1141 (90.3) | 0.008 | 3.6 | 1.3-9.9 |
| Sore throat | 42 (30.2) | 557 (44.1) | 0.002 | 0.5 | 0.4-0.8 |
| Dyspnea | 106 (76.3) | 121 (9.6) | <0.001 | 30.3 | 19.7-46.8 |
| Deterioration | 83 (59.7) | 446 (35.3) | <0.001 | 2.7 | 1.9-3.9 |
| Nasal congestion | 56/91 (61.5) | 680/846 (80.4) | <0.001 | 0.4 | 0.2-0.6 |
| At least one chronic disease | 97 (69.8) | 312 (24.7) | <0.001 | 7.0 | 4.8-10.3 |
| 3 or more chronic diseases | 58 (41.7) | 74 (5.9) | <0.001 | 11.5 | 7.6-17.4 |
| Cardiovascular disease | 65 (46.8) | 110 (8.7) | <0.001 | 9.2 | 6.3-13.5 |
| Chronic obstructive pulmonary disease | 17 (12.2) | 13 (1.0) | <0.001 | 13.4 | 6.4-28.2 |
| Asthma | 5 (3.6) | 31 (2.5) | 0.419 | 1.4 | 0.6-3.9 |
| Other chronic lung disease | 3 (2.2) | 9 (0.7) | 0.079 | 3.0 | 0.8-11.5 |
| Diabetes mellitus | 19 (13.7) | 53 (4.2) | <0.001 | 3.6 | 2.1-6.3 |
| Immune disease | 5 (3.6) | 34 (2.7) | 0.089 | 1.3 | 0.5-3.5 |
| Chronic kidney disease | 12 (8.6) | 27 (2.3) | 0.001 | 4.3 | 2.1-8.7 |
| Rheumatological disease | 5 (3.6) | 23 (1.8) | 0.312 | 2.0 | 0.8-5.4 |
| Neurological disease | 14 (10.1) | 33 (2.6) | <0.001 | 4.2 | 2.2-8.0 |
| Chronic liver disease | 6 (4.3) | 43 (3.4) | 0.112 | 1.3 | 0.5-3.1 |
| Cancer | 18 (12.9) | 29 (2.3) | <0.001 | 6.3 | 3.4-11.7 |
| Obesity | 32 (23.0) | 57 (4.5) | <0.001 | 6.4 | 3.9-10.2 |
| HIV infection | 3 (2.2) | 24 (1.9) | 0.834 | 1.1 | 0.3-3.8 |
| Other chronic disease | 24 (17.3) | 114 (9.0) | 0.002 | 2.1 | 1.3-3.4 |
| Onset of symptoms more than 3 days | 46 (33.1) | 216 (17.1) | <0.001 | 2.4 | 1.7-3.5 |
| Influenza vaccine | 8 (5.8) | 58 (4.6) | 0.539 | 1.2 | 0.6-2.7 |
| Influenza A(H1N1) | 59 (42.4) | 478 (37.8) | 0.289 | 1.2 | 0.8-1.7 |
| Influenza A(H3N2) | 48 (34.5) | 424 (33.6) | 0.823 | 1.1 | 0.7-1.5 |
| Influenza A not subtyped | 12 (9.3) | 91 (7.2) | 0.386 | 1.2 | 0.6-2.3 |
| Influenza B/Victoria | 18 (12.9) | 270 (21.4) | 0.019 | 0.5 | 0.3-0.9 |
| Co-infections | 12 (8.6) | 63 (5.0) | 0.070 | 1.8 | 0.9-3.4 |
| Respiratory syncytial virus | 6 (4.3) | 18 (1.4) | 0.013 | 3.1 | 1.2-8.0 |
| SARS-CoV-2 | 3/39 (7.7) | 13/382 (3.4) | 0.182 | 2.4 | 0.6-8.7 |
| Rhinovirus | 2/39 (5.1) | 16/382 (4.2) | 0.782 | 1.2 | 0.3-5.6 |

ARF – acute respiratory failure; OR – odds ratio; SARS-CoV-2 – severe acute respiratory syndrome coronavirus 2.
